# Copper deficiency myeloneuropathy in a patient with haemachromatosis: a case report

**DOI:** 10.4076/1757-1626-2-6168

**Published:** 2009-08-07

**Authors:** Cosmo Scurr, Barry Sampson, Joanna Ball, Carolyn Gabriel

**Affiliations:** 1 Department of Undergraduate Medicine, Imperial College LondonSW7 2AZUK; 2 Department of Clinical Chemistry, Charing Cross Hospital, Imperial Healthcare NHS TrustLondon, W6 8RFUK; 3 Department of Neurology, St Mary’s Hospital, Imperial Healthcare NHS TrustLondon, W2 1PDUK

## Abstract

A 64-year-old British Caucasian man presented with red skin wheals and breathlessness and then developed a progressive neurological syndrome. Investigation revealed hereditary haemachromatosis, porphyria, and a myelodysplastic syndrome. No unifying diagnosis was made, and his neurological symptoms remained unexplained, until further studies revealed an underlying copper deficiency.

## Case presentation

A 64-year-old British Caucasian man became unwell with a generalised rash, breathlessness and fatigue in late 1999, the day after returning from a golfing holiday in Palm Springs. He was noted to be suntanned, had an eczematous rash around the neck, and had generalised raised erythematous wheals. He had lost weight in recent weeks. He had only ever drunk alcohol in moderation and had smoked only briefly at school. He had no history of bowel disease or any previous surgery. There was no family history of neurological disease.

Examination at this stage was otherwise unremarkable.

Skin biopsy showed inflammatory changes only and he was treated symptomatically. Other investigations found raised inflammatory indices, an abnormal blood film with mild macrocytic anaemia and neutropenia, and increased liver transaminases. B12 and folate were normal. Bone marrow biopsy confirmed that both erythroid and myeloid cells lines were affected, but there were no chromosome or T cell abnormalities. A myelodysplastic syndrome was thought to be likely.

On liver ultrasound scan there was increased echogenicity and MRI suggested iron deposition. He had high serum ferritin and transferrin saturation, and a liver biopsy showed iron deposition and fibrosis. Despite a lack of family history, genetic testing gave a diagnosis of hereditary haemochromatosis (C282Y homozygote).

Whilst he was undergoing these investigations he developed neurological symptoms: aching below the knees and then gait unsteadiness. There was also numbness of the fingers, but this did not progress.

On neurological examination, there was a fine tremor without pseudoathetosis and a severely ataxic gait with a positive Romberg test. Cranial nerves were normal. There was mild wasting of the quadriceps and anterior calf muscles, but no wasting of the upper limbs. Tone and power were normal. Reflexes were present with crossed adductors. Pin-prick sensation was reduced to mid-thigh, light touch reduced to below the knees, proprioception was reduced to the hips, and vibration reduced to the costal margins. Nerve conduction studies showed a mild to moderate generalised sensorimotor axonal polyneuropathy, which was felt to be insufficient to fully explain his sensory ataxia. He had normal imaging of brain and spinal cord other than moderate cervical spondylosis without cord signal change. There was no lumbar root enhancement. Somatosensory evoked potentials were absent from the lower limbs. Cerebrospinal fluid was normal other than slightly raised protein at 0.7 g/dl.

Further investigation revealed a mildly raised homocysteine though normal urinary methylmalonic acid. Urinary porphyrins were raised and detailed urinary and faecal porphyrin studies indicated a defect at the level of uroporphyrinogen decarboxylase, consistent with a diagnosis of porphyria cutanea tarda.

Paraneoplastic disease was considered, but PET scan, whole body CT, upper and lower GI endoscopy were all normal. He was treated with B12 and has had this regularly since and also commenced regular venesection which has successfully normalised iron stores.

At this stage, this man with a profound sensory ataxia had accrued three new diagnoses of myelodysplasia, hereditary haemachromatosis and porphyria cutanea tarda; none of which explained his neurological phenotype. These could be linked only in that iron overload can precipitate porphyria cutanea tarda.

His neurological problems progressed to numbness below the knees and he developed further unsteadiness, requiring a stick to walk after one month and two sticks after two months. A year later he had developed extensor plantars, a very severe allodynia and painful paraesthesiae in the legs. He became wheelchair dependent by two years and continued to lose a total of 3 stones in weight.

At this stage, in 2001, he was re-investigated and was found to have a very low copper level at 0.37 μmol/L (reference range 12-20), even after reinitiating venesection, and a raised zinc level at 34.4 μmol/L (reference range 10-18). Caeruloplasmin was low at 0.02 g/l (reference range 0.2-0.4 g/L).

Copper excretion studies showed normal urinary copper excretion of < 0.1 μmol/day (reference range <1 μmol/day). Copper excretion after a penicillamine challenge increased to 1.20 μmol/day (normal subjects <25 μmol/day, Wilson Disease > 25 μmol/day), and there was a cyclic and inverse variation in serum copper and zinc concentrations with the copper level seeming to fall around the times of therapeutic phlebotomy.

He was given five days treatment with intravenous copper sulphate (2 mg/day), then oral copper sulphate (2 mg/day), and the neuropathic pains significantly abated and he was able to stand at the sink and in the shower. He continued oral copper sulphate for the next 18 months and remained stable.

## Discussion

Chronic sensory ataxia can occur secondary to inherited conditions (e.g. spinocerebellar ataxias, Friedrichs ataxia), infections (e.g. tabes dorsalis, HIV), vitamin deficiency (e.g. B12, folate, vitamin E), toxins (e.g. pyridoxine), paraneoplasia and immune syndromes (e.g. Sjogrens syndrome and inflammatory neuropathies). The underlying pathology can be in the sensory nerve, dorsal root ganglion, dorsal root, dorsal root entry zone or dorsal column of the spinal cord.

The first report of a copper deficiency myeloneuropathy in humans was published in 2001, shortly before our patient was investigated for the second time, in a female with previous partial gastrectomy [1]. A similar ataxic myelopathy had been described as Swayback disease in various animals for many years [2]. In Swayback disease, Wallerian degeneration occurs, characterised by demyelination and microcavitation especially in the white matter of the cervical cord. Recently, imaging studies in some human cases of copper deficiency have shown T2 hyperintensity in the dorsal cervical and thoracic cord [3].

Since then, a handful of case reports have been published, mostly in patients following gastrectomy [1,4-6], but even in these patients it is rare, and additional insults such as small bowel bacterial overgrowth have been postulated [7], presumably to exacerbate malabsorption. Cases can be associated with haematological problems and may or may not show MRI hyperintensities in brain and cord.

Zinc toxicity is another cause of copper deficiency [8]: Both copper and zinc are absorbed in the stomach and proximal duodenum. High zinc levels cause up-regulation of enterocyte metallothionein, a binding protein for both copper and zinc. Copper binds to metallothionein more avidly than zinc, remains in the enterocyte and is sloughed into the small bowel lumen, and thus not absorbed. It is also known that Clioquinol, a Cu/Zn chelating antibiotic used in Japan in the 1970’s, caused subacute myelo-optico-neuropathy [9].

## Conclusion

There are complex relationships between the trace elements that were abnormal in our patient and theoretically the pathophysiology could be explained by these. Haemachromatosis can cause raised gastrointestinal absorption of zinc leading to accumulation, which could potentially predispose to secondary Cu deficiency [8]. Although copper is bound to caeruloplasmin in serum, this is not exchangeable and caeruloplasmin is not a copper transport protein, but an oxidase. Caeruloplasmin oxidises ferrous ions and is thus necessary for supplying iron for haemoglobin formation. Caeruloplasmin is also involved in iron transport in that it oxidises apotransferrin to transferrin, and acaeruloplasminaemia causes iron toxicity because iron builds up in tissues. It is unlikely in our case that the hereditary haemachromatosis diagnosis is wrong, since iron stores responded to venesection, but low caeruloplasmin levels may have contributed to the iron overload syndrome and also to the development of the clinical copper deficiency syndrome.

Our hypothesis is that in this patient with relatively low caeruloplasmin levels, the development of haemachromatosis led to a copper deficiency myeloneuropathy, thus explaining all the features of this case.

## Abbreviations

CT: computed tomography
GI: gastrointestinal
MRI: magnetic resonance imaging
PET: positron emission tomography
